# Designing Soft Mobile Machines Enabled by Dielectric Elastomer Minimum Energy Structures

**DOI:** 10.3390/polym14071466

**Published:** 2022-04-04

**Authors:** Fan Liu, Ning An, Wenjie Sun, Jinxiong Zhou

**Affiliations:** 1 Xi’an Institute of Space Radio Technology, Xi’an 710100, China; kinter616@163.com; 2 School of Aeronautics and Astronautics, Sichuan University, Chengdu 610065, China; anning@scu.edu.cn; 3 School of Mechanical and Precision Instrument Engineering, Xi’an University of Technology, Xi’an 710048, China; 4 State Key Laboratory for Strength and Vibration of Mechanical Structures, Shaanxi Engineering Laboratory for Vibration Control of Aerospace Structures, School of Aerospace, Xi’an Jiaotong University, Xi’an 710049, China; jxzhouxx@mail.xjtu.edu.cn

**Keywords:** dielectric elastomer, soft machines, minimum energy structures, finite element

## Abstract

Dielectric elastomers (DE) are ideal electro-active polymers with large voltage-induced deformation for the design and realization of soft machines. Among the diversity of configurations of DE-based soft machines, dielectric elastomer minimum energy structures (DEMES) are unique due to their ease of fabrication, readiness to extend into multiple segments, and versatility of design configurations. Despite many successful demonstrations of DEMES actuators, these DEMES devices are limited to immobile use. We report several possible implementations of soft mobile machines through the combination of DEMES design, finite element simulation, and experiment. Our designs mimic the biomimetic locomotion of inchworms and marry complex components such as ratchet wheels with soft DEMES actuators. We even elucidate that buckling of DE can be harnessed to achieve asymmetric feet, which is otherwise realized via more complicated means. The examples presented here enrich DE devices’ design and provide valuable insights into the design and fabrication of soft machines that other soft-active materials enable. All the codes and files used in this paper can be downloaded from GitHub.

## 1. Introduction

To achieve high energy conversion and high transmission efficiency and meet the requirements for high precision and intelligent control, industrial machines are usually manufactured from hard materials such as metals, ceramics, plastics, and glass through precision machining or additive manufacturing. However, with the development of materials science, the definition of traditional machines has also changed. More and more advanced actuators and new materials are widely used in the design and manufacture of conventional industrial machines, such as magnetic actuators with strain and temperature monitoring based on magnetostrictive materials [1], ferrogels under specific stimuli [2], polymers with transitions [3], flexible magnitoelectronics [4], etc. In contrast, nature is composed of soft machines, including animals, plants, and even human beings. More recently, there has been an upsurge of interest in the design, manufacture, and control of soft machines [5,6,7]. Compared with their rigid counterparts, soft machines are made of compliant materials which enable more adaptivity, large deformation with more degrees of freedom, and a better human-machine interface. In addition, integrating soft active materials that can sense, actuate, compute, and communicate into soft machines makes them more sensitive and agile [5,6,7,8].

The design, manufacture, and realization of soft machines rely on soft smart materials, mainly elastomers with moduli in the range close to that of biological tissues. Examples of these stimulus-responsive elastomers include shape memory polymers, liquid crystal elastomers, hydrogels, ionic-polymer-metal-composites (IPMC), and dielectric elastomers (DE) [5,6,7,8,9,10,11,12,13,14]. Among them, DE is an ideal candidate due to its large deformation, fast and silent response, and high energy density [15,16,17,18,19,20]. Recently, an electrophoretic process has been developed to fabricate a unimorph nanocomposite DE with 13 times modulus difference; a high-speed lens motor designed with such material can achieve 40 times change in focal length [21].

Intensive efforts have been made to design a variety of DE transducers. On the DE actuators front, many configurations have been developed such as rolled, stacked, diaphragm, tube, push-pull, and minimum energy, to mention just a few examples [15,16,17,18,19,20]. Among these, the dielectric elastomer minimum energy structures (DEMES) actuators have attributed great attention due to their ease of fabrication, simplicity in working principles, and wonderful extensible property [22,23]. DEMES thus have been developed for single or multiple-segment grippers, petal-like actuators, oscillators, and mechanical translational mechanisms [22,23,24,25,26,27,28]. Meanwhile, the Euler-Lagrange energy method was used to study the nonlinear dynamic behavior of DEMES, and the results show that DEMES systems possess harmonic resonances and superharmonic and subharmonic resonances [29].

The working principle of DEMES is simple. Laminating a pre-stretched DE membrane on another relatively stiff thin plate forms a planar bilayer system that is unstable to any infinitesimal perturbation. The bi-layer system buckles to release the stretching energy stored in the DE membrane and retains a stable configuration that minimizes the total energy of the system, which explains why it is called DEMES. Subjected to an applied voltage, the DE membrane expands in area, causing the buckled bilayer to open to a distance. The opening and closing of DEMES constitute the working mechanism of DEMES based actuators.

So far, the DEMES based actuators are largely static in the sense that they do not exhibit self-locomotion, and the movement of these devices is manipulated through external forces. Here, we first demonstrate through numerical simulation that the opening and closing of DEMES can be utilized to design a locomotive soft machine that can ride and climb a rod. A pre-stretched DE membrane constrained by rigid frames would buckle if a large voltage were applied through the membrane, resulting in the so-called loss of tension instability which is often regarded as a nuisance to be avoided. For ease of fabrication and demonstration, the instability, generated by tuning the reversible formation of buckling, is harnessed to design asymmetric feet to switch between two contact states with different coefficients to realize the locomotion of the walking machine. To verify this design concept, we describe a mobile soft machine’s design, simulation, and experiment by marrying DEMES actuators with hard ratchet wheels.

## 2. Energy Minimization of DEMES

We briefly summarize the theoretical principle of energy minimization of DEMES [30,31]. Due to the intrinsic inhomogeneous buckling deformation of DEMES, we formulate the theory following the nonlinear field theory of deformable DE developed by Suo et al. [32,33]. The total energy of a DEMES unit consists of the bending energy of the frame, the stretching energy of the elastomer membrane, and the electrostatic energy of the DE. A material point marked by material coordinate X moves to a new position in the current configuration with coordinate x. The mapping of deformation is described by the deformation gradient $F=\frac{\partial x}{\partial X}$. The bending of the frame is infinitesimal, and the bending energy of the frame is assumed to be a function of curvature as

(1) $$W_{frame}=1/2EI\kappa^{2},$$

where EI is the bending stiffness of the frame and the curvature κ is typically a function of coordinate X.

We ignore the contribution of the DE membrane to the bending energy of the DE-frame bilayer and only consider its stretching energy and electrostatic energy. The DE membrane is assumed to be a neo-Hookean-like material with finite deformation and ideal dielectric property [32,33], and the summation of the stretching and electrostatic energies of DE is given by

(2) $$W_{DE}(C,\tilde{E})=\frac{\mu_{0}}{2}(I_{1}-3)+\frac{1}{2}\lambda_{0}{\left(\ln J\right)}^{2}-\frac{\varepsilon }{2}JC_{IJ}^{-1}{\tilde{E}}_{I}E_{J},$$

where $C=F^{T}F$ is the right Cauchy-Green tensor; $\tilde{E}$ is the nominal electrical field, $I_{1}$ and J are the first and the third invariants; $\mu_{0}$ and $\lambda_{0}$ are shear modulus and bulk modulus of DE, respectively, and ε is the permittivity of the DE. Note that when the DE-frame deforms, the curvature κ varies, and the deformation gradient F varies accordingly. Therefore, the right Cauchy-Green tensor C and thus the energy of DE is expressed implicitly as the functions of κ, i.e., $W_{DE}(C(\kappa ),\tilde{E})$.

The total energy of the DEMES is thus expressed as

(3) $$W_{total}(\kappa ,\tilde{E},)=1/2EI\kappa^{2}+W_{DE}(C(\kappa ),\tilde{E}).$$

Prior to the application of voltage, the DEMES system buckles to reach a state that minimizes only the bending energy of the frame and the stretching energy of the membrane. Upon combined mechanical and electrical loadings, the DEMES system varies curvature to attain a new equilibrium that minimizes the total energy given by Equation (3), which is dictated by the following condition,

(4) $$\frac{dW_{total}(\kappa ,\tilde{E},)}{d\kappa }=0.$$

In addition to Equation (4), the stress $S_{IK}(X)$ and the nominal electrical displacement ${\tilde{D}}_{K}(X)$ in DE obey

(5) $$\frac{\partial S_{IK}(X)}{\partial X_{K}}+B_{I}(X)=0,$$

and

(6) $$\frac{\partial {\tilde{D}}_{K}(X)}{\partial X_{K}}=Q(X).$$

Equations (5) and (6) are the mechanical and the electrical equilibrium equations in the volume and should be supplemented by the associated boundary conditions. B(X) and Q(X) are body force and the charge in the volume.

The PK2 stress of DE and the nominal electrical displacement are derived from the free energy DE as [34]

(7) $$S_{IJ}(C,\tilde{E})=2\frac{\partial W_{DE}(C,\tilde{E})}{\partial C_{IJ}}=\mu_{0}\delta_{IJ}+\lambda_{0}\ln JC_{IJ}^{-1}+\varepsilon J{\tilde{E}}_{K}{\tilde{E}}_{L}\left(C_{KI}^{-1}C_{LJ}^{-1}-\frac{1}{2}C_{KL}^{-1}C_{IJ}^{-1}\right),$$

and

(8) $${\tilde{D}}_{I}(C,\tilde{E})=-\frac{\partial W_{DE}(C,\tilde{E})}{\partial {\tilde{E}}_{I}}=\varepsilon J{\tilde{E}}_{I}C_{IJ}^{-1}.$$

In numerical calculation, the tangent moduli are crucial for the numerical implementation and are given as [34]

(9) $$H_{IJKL}=4\frac{\partial W_{DE}\left(C,\tilde{E}\right)}{\partial C_{IJ}\partial C_{KL}}=-\lambda_{0}\ln J(C_{IK}^{-1}C_{JL}^{-1}+C_{IL}^{-1}C_{JK}^{-1})+\lambda_{0}C_{IJ}^{-1}C_{KL}^{-1}-\varepsilon J{\tilde{E}}_{M}{\tilde{E}}_{N}\left[C_{MI}^{-1}(C_{NK}^{-1}C_{JL}^{-1}+C_{NL}^{-1}C_{JK}^{-1})+C_{NJ}^{-1}(C_{IK}^{-1}C_{ML}^{-1}+C_{IL}^{-1}C_{MK}^{-1})\right]\;+\varepsilon J{\tilde{E}}_{M}{\tilde{E}}_{N}\left[\frac{1}{2}C_{MN}^{-1}(C_{IK}^{-1}C_{JL}^{-1}+C_{IL}^{-1}C_{JK}^{-1})+C_{MK}^{-1}C_{NL}^{-1}C_{IJ}^{-1}+(C_{MI}^{-1}C_{NJ}^{-1}-\frac{1}{2}C_{MN}^{-1}C_{IJ}^{-1})C_{KL}^{-1}\right],$$

(10) $$e_{IJK}=2\frac{\partial W_{DE}(C,\tilde{E})}{\partial C_{JK}\partial {\tilde{E}}_{I}}=-\varepsilon J{\tilde{E}}_{L}\left(C_{KL}^{-1}C_{IJ}^{-1}-C_{KI}^{-1}C_{JL}^{-1}-C_{IL}^{-1}C_{JK}^{-1}\right),$$

(11) $$\varepsilon_{IJ}=-\frac{\partial W_{DE}(C,\tilde{E})}{\partial {\tilde{E}}_{K}\partial {\tilde{E}}_{L}}=\varepsilon JC_{IJ}^{-1}.$$

All these derivatives and tangents can be implemented and coded into homemade codes [34,35] or a user material subroutine (UMAT) by Zhao et al. [36]. We utilized the UMAT to perform our simulation by using ABAQUS 6.14. All the codes and files used in this paper can be downloaded from GitHub. https://github.com/XJTU-Zhou-group/FEM-soft-mobile-machines.

## 3. Design, Simulation, and Experiment of DEMES Soft Mobile Machines

**Figure 1:**
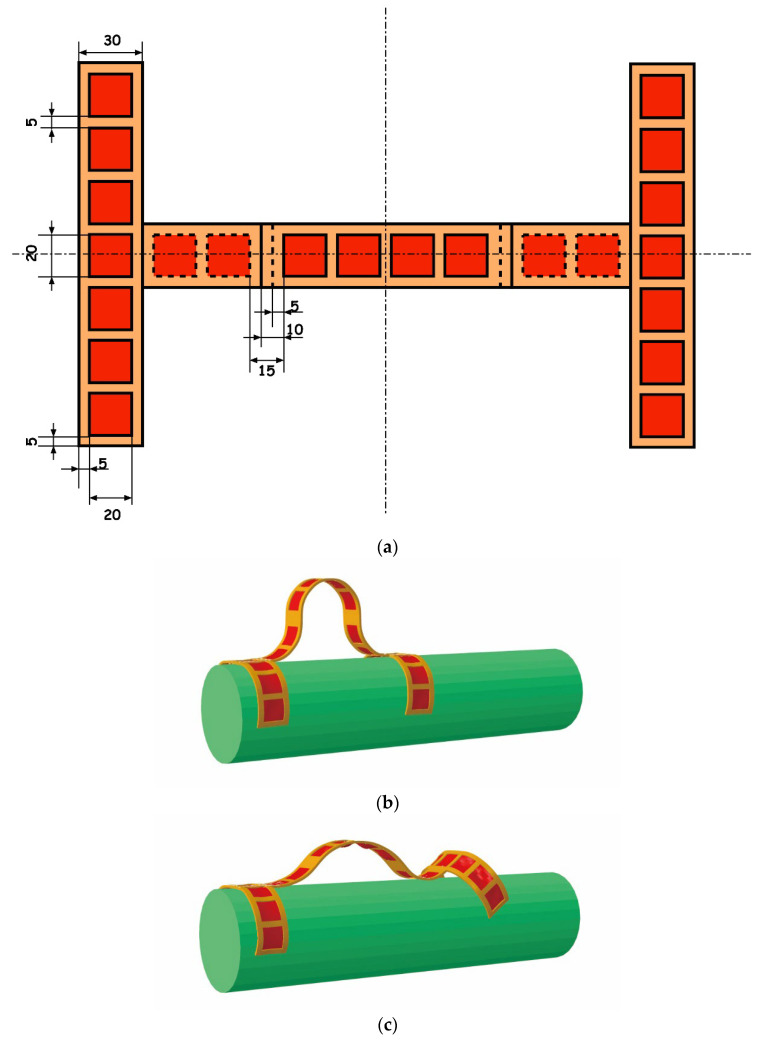

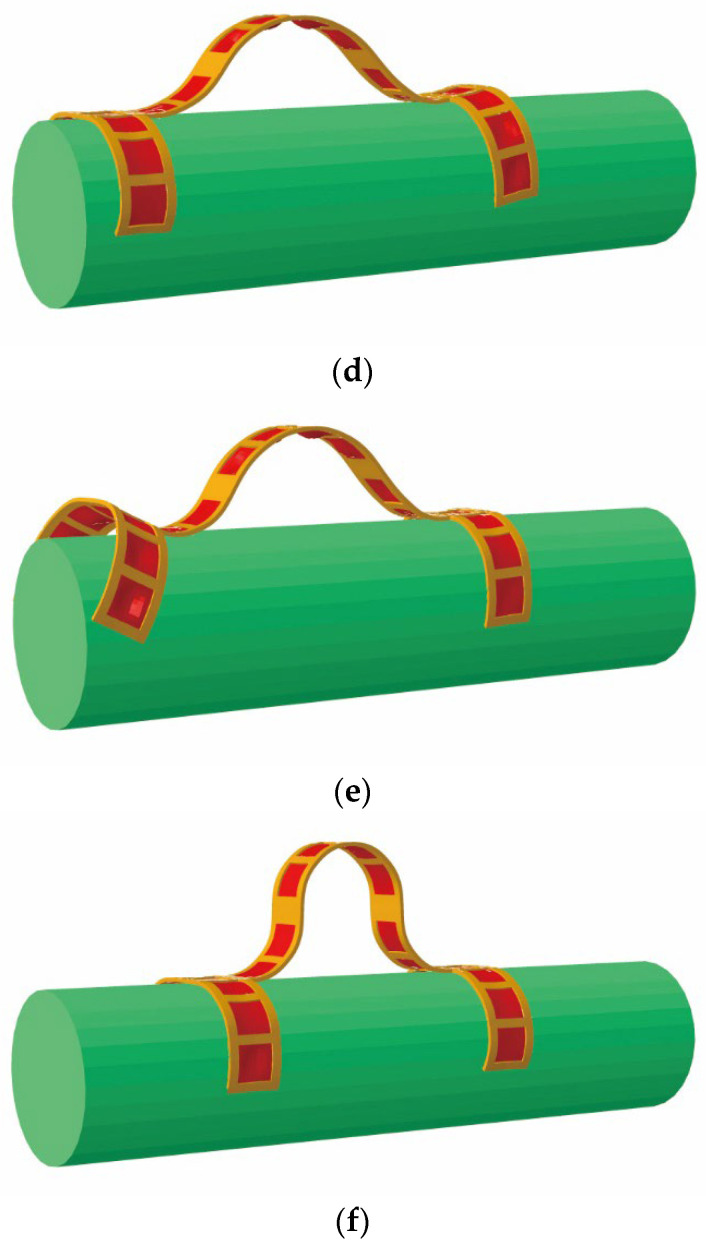
Design and simulated locomotion of an inchworm-inspired soft machine climbing a rod. (**a**) Planar view and dimensions (in millimeters) of the designed climber consisting of the anterior leg, hind leg, and central body. The central body has six segments of DEMES units and both the anterior and hind legs have seven units, with a red color DE membrane and yellow color frame. (**b**–**f**) A series of looping motions of the DEMES climber climbing a rod.

With the powerfulness of a commercial nonlinear FEM solver, ABAQUS, we designed and simulated three DEMES soft machines, and eventually experimentally demonstrated the locomotion of a DEMES soft machine. The first example presented here is a DEMES climber. Figure 1a shows the planar view of the designed DEMES structure and its dimensions. The DEMES climber consists of three main parts, an anterior leg, a hind leg, and a central body. Each part has multiple segments of DEMES units with pre-stretched DE marked in red color and frame marked in yellow. Figure 1b–f present a series of looping motions of the DEMES climber climbing a rod. In the unactuated state of the climber (b), the anterior leg, the hind leg, and the central body all buckle, and the climber harnesses the buckling to grasp the rod. The anterior leg is firstly actuated and unclasps the rod; actuation of the central body follows, and the expansion of the central body pushes the anterior leg forward (c). The actuation of the anterior leg is powered off and the rod is clasped again (d). Then, the hind leg is actuated and unclasps the rod (e). The actuation of the central body is powered off, pulling the hind leg forward. Then un-actuation of the hind leg follows, and the climber restores its original state with a forward locomotion distance (f). In the simulation given in Figure 1 and the following examples, we use the following parameters if otherwise explicitly stated. The material constants for DE are density 960 kg/m^3^, shear modulus $\mu_{0}$ = 22.5 kPa, permittivity ε = 3.717 × 10^−11^. The frame was modeled as linear isotropic material with Young’s modulus of 2.3 GPa and Poisson’s ratio of 0.394. The rod was assumed to be rigid with a very large modulus of 2 × 10^14^. The friction between the climber and the rod was accounted for and a friction coefficient of 0.3 was used in the simulation. When each DEMES unit was actuated, an applied electrical field, 2 × 10^7^ V/m, was ramped slowly to make the actuation a quasi-static process. Approximately 1.2 times of pre-stretch was used in the longitudinal direction of each DEMES unit.

**Figure 2:**
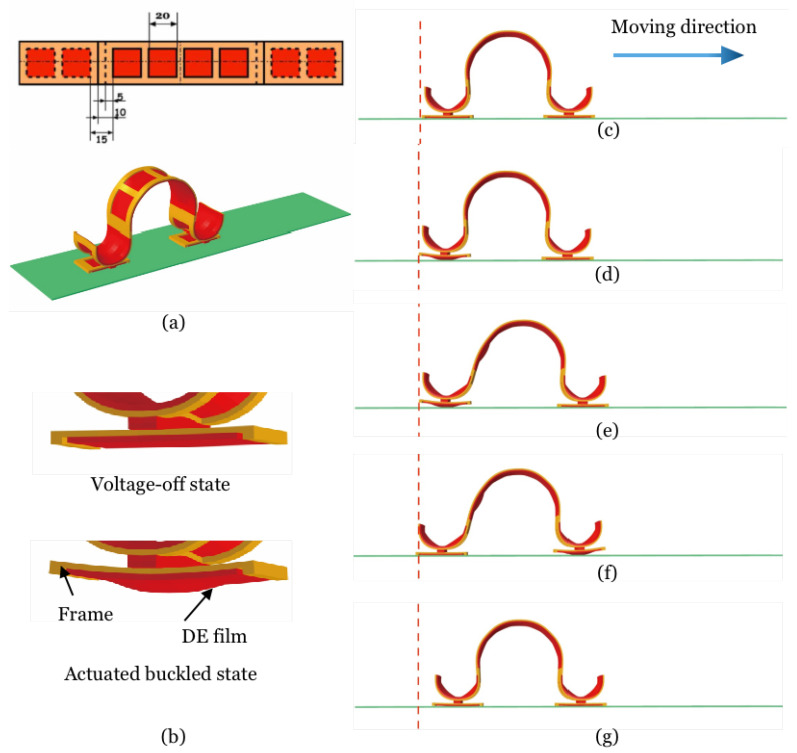
Design and modeling of a DEMES walker with switchable asymmetric feet realized by harnessing buckling instability. (**a**) Planar view and as-fabricated state of the DEMES walker. The central body consists of six DEMES units with a red color DE membrane and a yellow color frame. (**b**) Zoomed in picture of the voltage-off state (left) and actuated buckled state (right) of a foot for the walker. In the voltage-off state, the smooth frame is in contact with the ground, and a friction-free state is assumed, while in the buckled state the bulged DE membrane is in contact with the ground and a high friction coefficient is attained, realizing a foot design with switchable friction coefficients. (**c**–**g**) A series of actuations taken by the DEMES walker in order to realize locomotion.

Figure 2 gives the design and modeling of a DEMES walker with switchable asymmetric feet that enable unidirectional locomotion, which is the core of the design of a mobile soft robot. Previously, the unidirectional locomotion of a mobile soft robot was realized by the adoption of rachet wheels [37,38]. Here, we describe the realization of asymmetric friction by harnessing the buckling instability of DEMES. We assume the frame is made of friction-free hard material, while the DE, similar to the VHB type, is sticky and a high friction coefficient is assumed in contact with the ground. Figure 2a gives the design and the as-fabricated state of the walker. Figure 2b illustrates the zoomed in picture of the voltage-off state (left) and actuated buckled state (right) of a foot of the walker. In the voltage-off state, the smooth frame is in contact with the ground, while in the buckled state the bulged DE membrane is in contact with the ground, realizing a foot design with switchable friction coefficients. Figure 2c–g shows a series of actuations taken by the DEMES walker in order to realize locomotion. The hind foot of the walker in the original state (c) is actuated and attains a high friction contact with the ground (d); the central body is then actuated and expands and pushes the anterior foot forward (e); (f) follows where the anterior foot is actuated but the hind foot is switched to voltage-off state; the central body is switched off and contracts and pulls the hind foot forward, and eventually the walker attains its original state with a forward locomotion distance (g). The locomotion of the DEMES walker is realized through the combination of expansion and contraction of the central body DEMES unit and switching of friction states of the anterior and hind feet. Generating controllable reversible buckling in the foot unit is the key to the design. The parameters used for the simulation of the DEMES walker are the same as that given in the previous example except that different pre-stretches are taken for different parts of the walker: 1.2 for the central body and 1.3 for the feet. A friction coefficient of 0.3 was assumed for the DE-ground interface, while 0 was used for the frame-ground interface.

**Figure 3:**
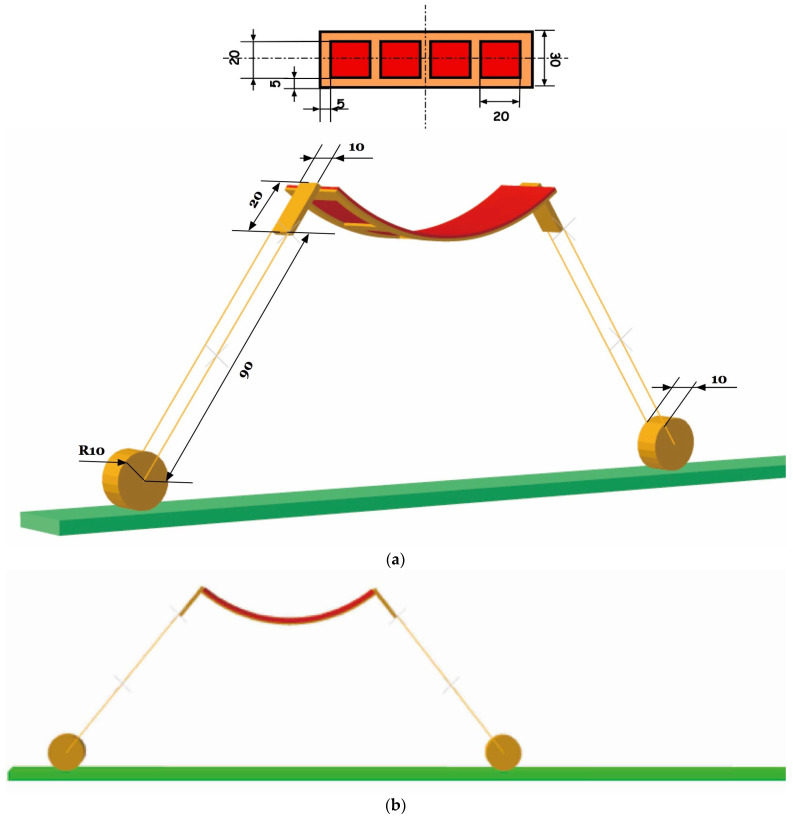

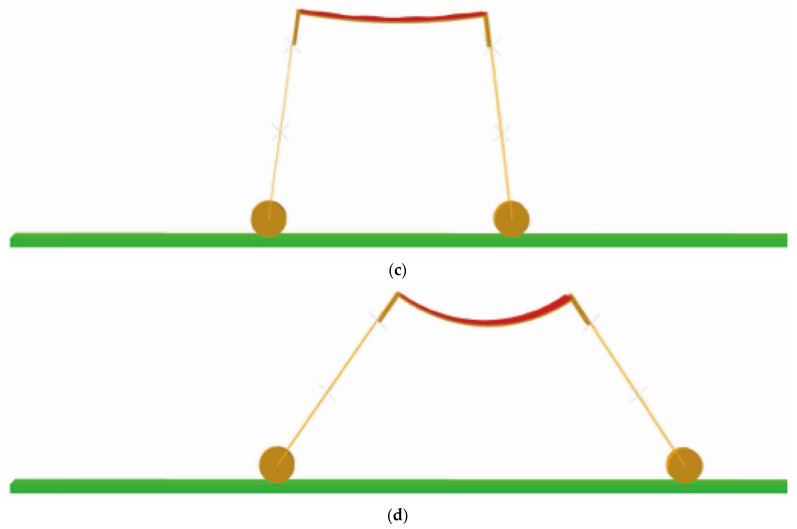
Design and modeling of a DEMES soft machine realized through the combination of a soft DEMES central body and hard legs and wheels. Different friction coefficients are assumed and switched during the process of locomotion. (**a**) Planar view and as-fabricated state of the soft machine. The central body buckles to minimize the total energy of the system. (**b**–**d**) A series of actuations and motions are taken by the soft machine for forward locomotion.

We then come to the design of a soft machine enabled by combining hard wheels and a soft DEMES actuator. Similar ideas of integrating hard mechanical components with soft DE actuators are proposed by the authors [35,36]. Figure 3a shows the planar view and as-fabricated state of the soft machine. The central body buckles to minimize the total energy of the system. Figure 3b–d shows the simulation of a series of actuations and motions taken by the soft machine for forward locomotion. Original state (b); The contact of the anterior wheel with the ground assumes a friction coefficient of 0.3 while 0 is assumed between the hind wheel and the ground. Actuation of the central body expands the DEMES structure and the hind wheel rolls forward (c); the friction coefficients between the anterior and hind wheels and the ground are switched, and the powering-off of the central body makes it contract and the anterior wheel rolls forward (d). The modeling of this hybrid machine is valuable since most of the soft machines developed so far have both hard and soft components. It is, nevertheless, challenging since it incorporates soft deformable DEMES unit and wheels with rigid motion and contacts. This is a typical rigid-flexible coupling simulation and we implement it by using the dynamic/implicit algorithm in ABAQUS.

**Figure 4:**
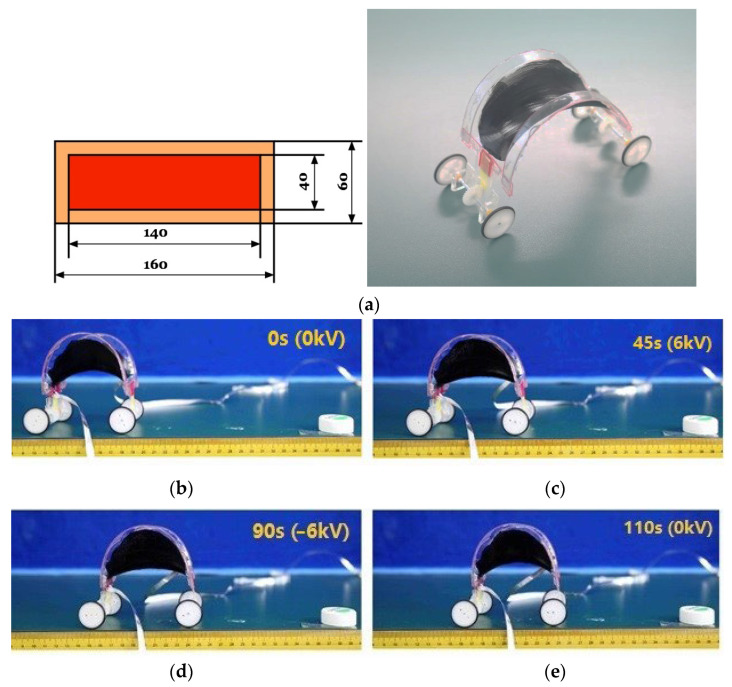
Design and experiment of a soft machine enabled by the combination of soft DEMES and hard ratchet-wheels. (**a**) Planar view and as-fabricated state of the DEMES. (**b**–**e**) A series of still frames from the experimental video shows the locomotion of the soft machine.

Finally, we experimentally demonstrate a prototype of a soft machine driven by a DEMES actuator. A piece of a rectangular DE membrane is pre-stretched five times along the length direction and fixed to a hard PMMA frame. The DE-frame bilayer buckles and forms a saddle-shaped DEMES actuator. The DEMES actuator is integrated with ratchet wheels, resulting in a soft machine undergoing unidirectional locomotion. Figure 4a gives the dimensions of the DEMES actuator and the assembled soft machine. Figure 4b–e presents a series of still frames from the experimental video showing the locomotion of the soft machine. A triangle wave voltage with a period of 20 s and a peak voltage of 6 kV was applied to drive the soft machine. An average velocity of about 0.53 mm/s was estimated from the experimental video.

## 4. Concluding Remarks

When a piece of soft DE membrane is pre-stretched and laminated on a hard frame and then set free, the DE-frame bilayer buckles to release the elastic energy stored in the bilayer, provided the frame is made of materials that are not too rigid and can be deformed by DE. The DE-frame bilayer may morph from its buckling state and attain a new state to minimize the total energy, resulting in a DEMES structure that can work as a soft actuator for various applications. DEMES actuators are attractive in the community of electro-active polymers since they are easy to fabricate, simple to extend into multiple segments, and versatile for configuration design.

To date, DEMES actuators are used for immobile devices such as grippers and bending actuators. We describe the use of DEMES structures for the design and fabrication of mobile soft machines through a combination of numerical simulation and experiment. Designs of the soft machines presented here include an inchworm-inspired climber, a walker with asymmetric feet, and a mobile machine enabled by wheels and DEMES. We finally experimentally demonstrate a unidirectionally locomotive machine by integrating soft DEMES and hard ratchet wheels. For the design of the DEMES walker, asymmetric feet with switchable coefficients are realized by harnessing the buckling instability of DE. Implementation of asymmetric feet via this novel mechanism is quite simple and avoids the complexity of other means. Our numerical and experimental design strategy can be developed to design and fabricate DE-based soft machines and shed light on the invention of soft machines based on other soft active materials. Those results also can provide new ideas and technical means for aerospace and biomedical fields such as intelligent deformable wings, space intelligent grippers, and human body micro-robots.

## Data Availability

https://github.com/XJTU-Zhou-group/FEM-soft-mobile-machines.
